# Risk of PTSD Due to the COVID-19 Pandemic Among Patients in Opioid Substitution Treatment

**DOI:** 10.3389/fpsyt.2021.729460

**Published:** 2021-09-30

**Authors:** Isabella Fuchs-Leitner, Kurosch Yazdi, Nikolas W. Gerstgrasser, Matthias G. Tholen, Sophie-Therés Graffius, Alexander Schorb, Jan Rosenleitner

**Affiliations:** ^1^Medical Faculty, Johannes Kepler University Linz, Linz, Austria; ^2^Department of Psychiatry - Specialization Addiction Medicine, Kepler University Hospital GmbH, Linz, Austria; ^3^University Hospital of Psychiatry, Psychotherapy and Psychosomatics, Paracelsus Medical University, Salzburg, Austria

**Keywords:** COVID-19, drug use disorder, opioid substitution therapy (OST), PTSD, IES-R, DASS-21

## Abstract

**Background:** The impact of the COVID-19 pandemic on the mental health of patients suffering from addictive disorders is of major concern. This study aimed to explore the presence and potential increase in post-traumatic stress disorder (PTSD) symptoms, depression, and anxiety since the beginning of the pandemic for patients in opioid substitution therapy (OST).

**Methods:** This cross-sectional survey study evaluated a clinical sample of patients in OST (*N* = 123). Symptoms of post-traumatic stress disorder (PTSD) due to the COVID-19 pandemic were assessed by an adapted version of the impact of event scale (IES-R), resulting in two subgroups of low and high risk for PTSD. The depression, anxiety, and stress scale (DASS-21) was applied to collect data on the respective symptoms, and changes since the onset of the pandemic were reported on separate scales. Sociodemographic and COVID-19 related factors, as well as data on craving, consumption patterns, concomitant use, and the drug market were further assessed.

**Results:** A binary logistic regression analysis confirmed the impact of self-perceived higher burden by psychological and economic factors on the elevated risk for PTSD due to the pandemic. The high-risk PTSD group also showed higher levels of depression, anxiety and stress, as well as a more pronounced deterioration in these symptoms since the pandemic. While reported levels of craving did not differ between the two groups, the high-risk PTSD group indicated a significantly higher increase in craving since the crisis, when compared to the low-risk group.

**Discussion:** Our findings demonstrate elevated levels of clinical symptoms among patients in OST, with more than a quarter of patients found at risk for PTSD due to the COVID-19 pandemic. Furthermore, about 30–50% of our patients reported concerning levels of depression, anxiety, or stress. Special attention should be drawn to these findings, and potential deterioration of the situation should be addressed by health care facilities. Particularly, psychological, and financial burden due to the crisis were identified as factors increasing the risk for PTSD. These factors can easily be evaluated during routine anamneses, and might be a valuable source of information, when special attention is needed.

## Introduction

The effects of the COVID-19 pandemic influence our daily lives in many aspects since the outbreak in Wuhan at the end of 2019. Negative consequences are exacerbated by social distancing, fear of infection, lockdowns, travel restrictions, unemployment due to the crisis, as well as uncertainty of the future. In respect to mental health of the general population, the COVID-19 pandemic is expected to promote development and deterioration of mental and behavioral disorders, and potentially increase a variety of clinical symptoms including depression, anxiety, denial, fear or sleep disorder (1, 2). Furthermore, lockdowns and quarantine promote additional psychological stressors (3). In Austria, a study found an increase in depression rates between the time before and after the first lockdown in 2020. The most pronounced negative effect on developing depressive symptoms was identified as a combination of higher levels of stress and stronger perceived loneliness during lockdown (4). The challenges of the pandemic could additionally result in an increase in addictive behaviors and SUDs as maladaptive coping strategies (5).

### COVID-19 Related Factors and Substance Use Disorders

The negative consequences of the COVID-19 pandemic include physiological, psychological, social and economic burdens [see also our prior research on alcohol use disorder (6), as well as a perspective based on a small sample of patients in treatment for drug use disorder (7)]. These far-ranging effects might be particularly demanding for vulnerable groups, like patients suffering from substance use disorders (SUDs) (8). Serious implications for this subgroup including long-term socioeconomic and public health effects can be anticipated (9). In particular, increased risk of infection and severity of COVID-19 symptoms, psychological stress and reduced access to addiction treatment services are of major concern.

From a *physiological* perspective, substance use disorders (SUDs) were found to increase the risk to contract COVID-19 (10). Persons suffering from drug use disorder often develop conditions regarding the respiratory system from inhalation drugs. An overall impaired immune system as well as damaging effects of drug use on the cardiovascular system further increase the risk of mortality associated with COVID-19 (11). As patients suffering from SUDs are at higher risk for COVID-19 and worse outcomes, individual worries about the physiological effects of the pandemic could be anticipated.

Demanding *psychological* aspects of the pandemic and lockdowns are evident. Major psychological stressors are driven by trauma exposure, levels of perceived stress and isolation, rendering risk factors for a deterioration of symptoms of depression and anxiety (12). An Italian study investigating psychopathological burden during the beginning of the pandemic found relatively high rates of depression, anxiety, irritability, and post-traumatic stress symptoms among patients with SUDs (13).

Negative *economic* effects are clearly anticipated, since global economy is struggling heavily with the financial consequences of the pandemic. Loss of income due to reduced working hours, or even job loss due to the pandemic represent major economic stressors on the individual level, and might be a source for further psychological burden (14). Lower perceived economic stability additionally promoted the risk of post-traumatic stress symptoms during the pandemic (15). Income reduction further elevates the risk for depression and anxiety (12).

*Social* interactions have been heavily restricted during the pandemic due to lockdowns and other government measures. In Austria, social life was interrupted by closure of bars and restaurants, and a ban on large public gatherings. Even social interactions in private parts of life had to be immensely reduced, and restrictions on non-essential movement (except medical care, food shopping, or exercise) further promoted isolation during the second wave of the pandemic. Taking this situation into account, a tremendous burden on patients with SUD stemming from reduced social support as a protective factor (16) could be expected. Since substance use often occurs in social contexts, a decrease in consumption for recreational users might be observed during lockdowns. However, regular substance use and more severe SUDs might probably increase (12).

### Psychopathological Symptoms Among Patients With SUDs, and During COVID-19: PTSD, Depression, Anxiety, and Stress

Already before the pandemic, high rates of post-traumatic stress disorder (PTSD) among patients with SUDs, but a low detection rate in treatment settings was assumed (17). In general, PTSD follows traumatic events and is characterized by a symptom pattern of intrusions, avoidance of thoughts and behaviors, negative changes in thoughts and mood, and changes in arousal and reactivity (18). Prior clinical research also confirmed relatively high rates of comorbid affective and anxiety disorders among patients in treatment for SUDs—a subgroup, which might also be characterized by a higher severity of this disorder (19). Furthermore, a complex interplay between psychiatric comorbidities and substance use is assumed. Among patients with opioid use disorder (OUD) depression has been identified as highly prevalent, and its impact on therapy outcome is anticipated, but poorly understood yet [for a recent review see Ghabrash et al. (20)]. The potential interplay between stress and risk for drug use was investigated among a sample of patients with OUD (21). Higher reported levels of stress have already been associated with early drop-out (22).

A rise in PTSD, anxiety and depression symptoms during the pandemic have been anticipated and confirmed in the general population (3, 23, 24). PTSD due to COVID-19 was expected as a common psychiatric response to the current pandemic and its related psychological stressors (25). Studies conducted in China and Italy during the initial stage of the pandemic, which were heavily affected areas, reported high rates of PTSD and psychological distress in the general population (26, 27). For patients with SUDs during this ongoing pandemic, negative mental health consequences including higher levels of depression, anxiety, irritability, and post-traumatic stress symptoms have already been confirmed (10, 12, 26). The COVID-19 pandemic renders an additional major source of distress for patients in opioid substitution therapy (OST), who are already more vulnerable in respect to their mental and social health. Close monitoring of this subgroup and providing stable OST services for this population is therefore mandatory during this crisis (28).

### Opioid Substitution Therapy (OST) and Concomitant Use of Illicit Drugs

Misuse of the OST medication (29) and concomitant use of other illicit drugs is highly prevalent, and therefore a major issue of concern in OST. A Swiss registry-based study, which was conducted before the pandemic (30) found that more than a third of all participants reported at least one cocaine consumption day in the past month. Furthermore, a positive association between the dosage of methadone and concomitant use of cocaine was observed. Australian patients receiving OST had a significant reduction in the depression subscale of the DASS-21 after 3 months of treatment, less pronounced improvements were seen in the stress and anxiety subscales (31). Compared to normative values patients in methadone maintenance treatment had higher stress, post-traumatic stress symptoms and cortisol levels (32). Data from an US-study showed, that patients, dropping out from OST, reported higher levels of stress, heroin- and cocaine-craving than participants, who stayed in OST during the observation time (22).

In the context of the pandemic, in our previous study (*N* = 32) 79% of the participants in OST indicated concomitant use of other illicit drugs during the initial phase of the pandemic (7). However, this number has to be interpreted with caution, given potential biases due to the small sample size and a high proportion of inpatient participants in this study. Developments on illicit drug markets due to the pandemic, as well as their direct and indirect consequences remain unclear. Due to government control strategies and border closures major interruptions in illegal drug supply were expected (33). Unavailability of substances could lead to hazardous activities, including self-manufacturing of substances or even a rise in cases of suicide (34). Increase in pricing and disruption of illicit opioids could have further severe impacts on the drug-taking community, including more cases of overdose (11). This risk is heightened by the consumption of other opioids than normally administered due to the lack of availability, as well as by accompanied changes in quality and strength of those substances (28, 35). Furthermore, social distancing may increase the probability of fatal overdoses due to isolation without opportunity for rescue (36, 37). Consequently, the situation on the drug market should be closely monitored, enabling reactions to further potentially negative implications for patients suffering from drug use disorder.

### Aims and Research Questions

Original data on patients suffering from drug use disorder, including those in OST during this ongoing pandemic are still sparse. Taking findings of studies focusing on SUDs in general (13) into account, an elevated risk to develop PTSD symptoms as a result of the crisis might be expected, and has to be monitored in this vulnerable group. Therefore, the main goal of the current study was to assess the presence and severity of PTSD symptoms due to the COVID-19 pandemic. To that end, PTSD symptoms were evaluated using an adapted version of the IES-R (38). The sample of patients in OST was accordingly split into two subgroups labeled as low or high risk for PTSD due to the pandemic based on the IES-R (but not as a professional diagnosis of PTSD). In this context, the impact of potentially contributing sociodemographic and various COVID-19 related worries and fears for different areas of life (physiological, psychological, economic and social factors) were investigated. Furthermore, levels of severity in psychopathology (depression, anxiety, and stress), as well as differences and changes on these measures since the beginning of the pandemic were evaluated between the two groups. Additionally, momentary craving, concomitant use of illicit substances, and developments on the Austrian drug market were assessed.

## Materials and Methods

### Participants and Procedure

For this cross-sectional survey study, data was collected from patients receiving treatment at two outpatient facilities in Austria. The duration of the study was 14 weeks, between end of November 2020 and beginning of March 2021. Only patients, who were currently in OST, and provided responses on nearly all of the items of the survey (defined as a maximum of four missing responses on the scales) were included in the final analysis, resulting in a total sample of *N* = 123. This study was conducted in accordance with the Declaration of Helsinki and approved by the local ethics committee. Participants provided written informed consent, and data was processed and analyzed anonymously. Data collection started after a new increase of COVID-19 incidence in Austria—also called the *second wave*—between December 2020 and February 2021. During this time period, hotels, restaurants, and bars remained closed, and social interactions were restricted in public and private areas of life by government measures.

### Survey Structure

#### Sociodemographic Data

Relevant sociodemographic variables were collected, including age, gender, employment, and relationship status.

#### Drug Consumption and Craving

Levels of drug consumption were assessed using the four items of the DUDIT-C (39) [Drug Use Disorder Identification Test (40)—consumption part]. Participants also indicated subjective momentary craving (on a Likert-scale from 0 to 10). Changes in craving and consumption patterns (i.e., frequency and quantity) were assessed on separate scales (ranging from −5 to +5).

#### Concomitant Use and Drug Market

Participants reported the use of other substances than prescribed. Addressing the Austrian drug market, changes in availability, pricing, and quality since the beginning of the pandemic were evaluated.

#### Impact of Event Scale–Revised (IES-R)

The IES-R (38) is commonly used as a screening measure to evaluate the presence and severity of PTSD symptoms. The scale was adapted to solely focus on the impact of the COVID-19 pandemic on PTSD symptoms, similarly to Vanaken et al. (16). To that end, the instruction and items were rephrased to clarify that all questions in this survey were assessing the effect of the pandemic, and no prior or other traumatic event. This measure consisted of three subscales, assessing PTSD symptoms of intrusion, avoidance, and hyperarousal. The German version of the IES-R presented good validity and reliability (79–90%) in the assessment of the psychological impact of traumatic events (41). A study evaluating a sample of participants with SUD reported good psychometric properties of the IES-R and its subscales (42): high internal consistency was found for the total score (Cronbach’s α = 0.95), as well as for all three subscale scores (Intrusion α = 0.92; Avoidance α = 0.85; Hyperarousal α = 0.91).

#### Depression, Anxiety, and Stress Scale (DASS-21)

The German version of the DASS-21 (43, 44) was used to evaluate self-reported clinical symptoms of depression, anxiety, and stress on three different subscales. The total score determines an overall level of burden as indicated by the participants. Again, changes on the different subscales since the beginning of the pandemic were assessed on separate scales (from −5 to +5). Good validity and reliability (78–91%) of the German version of the DASS-21 was found in previous studies in evaluating levels of depression, anxiety, and stress (43, 45). The IES-R and DASS-21 have both been used and validated in recent studies on the psychological impact of the COVID-19 pandemic (24, 41).

#### COVID-19 Factors

This assessment addressed worries and fears about four different areas of life: physiological, psychological, economic, and social factors. Participants were asked to think about the consequences of the pandemic and related government measures on their personal life. Subsequently, their perceived negative impact of the pandemic was assessed with one item per COVID-19 factor. To that end, participants were given examples of potential fears regarding the different areas of life, and asked to rate their worries on a Likert-Scale from 0 (no worries at all) to 10 (a lot of worries). *Physiological factors* included the fear to contract COVID-19, worries about other possible health problems in the context of COVID-19, restricted access to the health care system due to the pandemic, as well as postponed medical procedures. *Psychological factors* assessed negative feelings due to the pandemic like depression, anger, worries or helplessness. *Economic factors* addressed the negative financial consequences of the pandemic, such as job loss or the fear to lose one’s livelihood. *Social factors* focused on the negative impact on social life, like experiencing loneliness or isolation during lockdowns, as well as restrictions for many social interactions due to related government measures.

### Statistical Analysis

Data was analyzed using IBM SPSS Statistics for Windows (Version 25.0) (46). Descriptive statistics of the variables are reported in Table 1. The IES-R was adapted to assess PTSD symptoms exclusively for the COVID-19 pandemic (and no other traumatic events). Main analyses of this study were based on the cutoff score for being at risk of PTSD according to the IES-R total score. To that end, the total sample was split into two subgroups of patients indicating low or high risk of PTSD due to the pandemic [for more details see section Impact of Event Scale (IES-R) Adapted for COVID-19 below].

**Table 1:** Descriptive statistics for all variables in the total sample and the two subgroups, respectively.

	**Total** **(***N*** = 123)**	**Low IES-R** **(***N*** = 90)**	**High IES-R** **(***N*** = 33)**
	**Mean (SD)/Percent**	**Mean (SD)/Percent**	**Mean (SD)/Percent**
**IES-R adapted for COVID-19**			
Total score [0–88]	16.5 (13.5)	9.7 (6.6)	35.1 (9.3)
Intrusion scale [0–32]	4.8 (4.9)	2.5 (2.6)	11.0 (4.1)
Avoidance scale [0–32]	7.9 (6.3)	5.1 (4.0)	15.5 (4.7)
Hyperarousal scale [0–24]	3.8 (4.0)	2.1 (2.2)	8.6 (4.0)
**Sociodemographic factors**			
Age [in years]	38.5 (11.1)	38.6 (11.2)	38.4 (11.2)
Gender: Male	79.7%	78.9%	81.8%
Living alone: Yes	52.8%	51.1%	57.6%
Relationship: Yes	35.8%	36.7%	33.3%
Employment: Yes	43.1%	41.1%	48.5%
**COVID-19 factors [all scales from 0 to 10]**			
Physiological factors	3.2 (2.8)	2.8 (2.7)	4.4 (2.8)
Psychological factors	3.9 (3.2)	3.3 (3.0)	5.6 (3.2)
Economic factors	3.5 (3.3)	3.0 (3.1)	5.1 (3.6)
Social factors	3.4 (3.3)	2.8 (3.2)	4.9 (3.3)

*SD, standard deviation; IES-R, impact of event scale–revised*.

To evaluate the potential impact of sociodemographic variables and different COVID-19 factors on the risk of PTSD due to the pandemic, a binary logistic regression analysis was conducted. Furthermore, differences between the low- (*N* = 90) and high-risk (*N* = 33) PTSD-groups were evaluated for clinical symptoms (depression, anxiety, and stress) as well as for craving, using Mann Whitney tests. Changes on the symptomatology were assessed, and differences between the groups were further investigated. Findings on concomitant use of other illicit substances, and developments on the Austrian drug market are reported in a descriptive manner. Effect sizes for the different analyses are reported as correlation coefficient *r* and interpreted according to Cohen (47) as small (0.1–0.3), moderate (0.3–0.5), and strong (>0.5) effects.

## Results

### Descriptive Statistics

Descriptive statistics for the IES-R, sociodemographic variables, and COVID-19 factors are displayed for the total sample and the two subgroups (low- and high-risk PTSD) in Table 1.

### Impact of Event Scale (IES-R) Adapted for COVID-19

The IES-R (38) was adapted to evaluated PTSD symptoms due to COVID-19. In this sample, excellent internal consistency was found for the total IES-R score (Cronbach’s α = 0.91), and moderate to high levels for the three subscale scores (Intrusion α = 0.78; Avoidance α = 0.82; Hyperarousal α = 0.76). Rash et al. (42) examined a range of cutoff scores for the IES-R for suitability with a substance dependent sample. Their results indicated a recommended cutoff score of 22–24 on this scale to determine an elevated risk of PTSD. Cutoff values of 22–24 in this study met the goal to maximize sensitivity (92%, specificity of 57%), with an overall correct classification rate of PTSD cases of 77%. Based on these findings, a cutoff value of 24 was selected for this study. Accordingly, the total sample was split into two subgroups of patients indicating low (i.e., total IES-R score <24) or high risk of PTSD (i.e., total score ≥24) due to the pandemic. As confirmed by Mann-Whitney tests, the two subgroups did not only differ significantly in the total score of the IES-R (*z* = −8.5, *p* < 0.001), but also on all three subscales for intrusion (*z* = −7.9), avoidance (*z* = −7.6), and hyperarousal (*z* = −7.3, all *p*s < 0.001, all *r*s > 0.65; see descriptive data in Table 1).

### Modeling and Predicting Low and High-risk of PTSD Symptoms Due to COVID-19 With Logistic Regression Analysis

A binary variable was constructed for patients at low-risk (value = 0; *N* = 90) or high-risk (value = 1; *N* = 33) for PTSD due to the COVID-19 pandemic according to IES-R scores. A binary logistic regression analysis was then performed to investigate potential risk factors for PTSD. The model allows to evaluate the effects of sociodemographic factors (age, gender, living alone, and employment) and COVID-19 impact (physiological, psychological, economic, and social factors) on the probability of experiencing PTSD symptoms due to COVID-19. A backward variable selection procedure (Wald) was performed using a cutoff value of 0.27 (i.e., the proportion of patients with high-risk for PTSD in the total sample). Results of this regression analysis are presented in Table 2, for the initial model as well as for the final model after variable selection.

**Table 2:** Results of the binary logistic regression model for patients with high (vs. low) risk of COVID-19 related PTSD symptoms (according to a cutoff score of 24 in the IES-R).

	**B**	**SE**	**Wald χ^2^**	**OR**	**95% CI**	***p***
**Initial model (Step 1)**						
Age	−0.06	0.23	0.08	0.99	0.95–1.04	0.783
Gender	−0.21	0.59	0.13	0.81	0.25–2.58	0.723
Living alone	0.82	0.53	0.02	1.09	0.38–3.09	0.878
Relationship	−0.51	0.55	0.01	0.95	0.32–2.80	0.926
Employment	0.51	0.48	1.05	1.65	0.63–4.31	0.306
Physiological factors	0.68	0.09	0.58	1.07	0.90–1.28	0.445
Psychological factors	0.15	0.08	3.31	1.16	0.99–1.37	0.069
Economic factors	0.11	0.07	2.44	1.12	0.97–1.29	0.118
Social factors	0.11	0.07	2.11	1.11	0.96–1.29	0.146
Constant	−2.75	1.02	7,30	0.06		0.007
**Final model (Step 8)**						
Age	*	*	*	*	*	*
Gender	*	*	*	*	*	*
Living alone	*	*	*	*	*	*
Relationship	*	*	*	*	*	*
Employment	*	*	*	*	*	*
Physiological factors	*	*	*	*	*	*
**Psychological factors**	0.20	0.07	7.18	1.22	1.05–1.41	**0.007**
**Economic factors**	0.14	0.07	4.00	1.14	1.00–1.31	**0.045**
Social factors	*	*	*	*	*	*
Constant	−2.41	0.46	27.72	0.09		0.000

*Results and test statistics for the initial and final logistic regression model (step 8) are displayed. Significant results with p < 0.05 are presented in bold letters. SE, standard error; OR, odds ratio; CI, confidence interval*.

* *Variables dropped in backward selection procedure*.

The final model (step 8 with a correct classification rate of 0.71) included psychological and economic COVID-19 factors as predictors, and was statistically significant, χ^2^(2) = 17.1, *p* < 0.001. Nagelkerke *R*^2^ of 18.9% showed a moderate goodness of fit of the model with moderate to high levels of sensitivity (0.64) and specificity (0.73). Patients indicating a stronger negative impact by psychological COVID-19 factors had a higher risk (odds ratio of 1.21, *p* = 0.007) for PTSD. Economic COVID-19 factors (odds ratio of 1.14, *p* = 0.045) also increased the probability for PTSD according to IES-R scores.

### Depression, Anxiety, Stress (DASS-21), and Craving

The DASS was originally constructed to measure self-reported negative emotional states of depression, anxiety and stress, including 42 items (48). The short version DASS-21 (44) consists of 21 items (ranging from 0 to 3) with seven items per subscale. In the current study, the sum of all item scores was calculated for the total score (ranging from 0 to 63). For the subscores of depression, anxiety and stress the item scores of the respective subscales were summed, respectively^1^. For this measure, levels of severity and respective cutoff scores for the subscales were adapted from the original DASS (48). High internal consistency was found for the DASS-21 total score (Cronbach’s α = 0.95), and the three subscale scores (Depression α = 0.92; Anxiety α = 0.82; Stress α = 0.89).

#### Severity Levels of Depression, Anxiety, and Stress

Descriptive statistics of the DASS-21 and frequencies for the different levels of severity in the total sample, as well as for the two PTSD risk-groups are displayed in Table 3. Prevalence of depressive symptoms was particularly high in our sample with only half of the patients (52.8%) indicating normal severity levels on this subscale. Furthermore, symptoms of anxiety and stress were above the normal level for approximately a third of the patients in OST. Binary variables were created for the subscales indicating either normal or mild (0) or increased levels of severity (1 for moderate, severe, and extremely severe). Qui-square tests between these variables and the PTSD-risk groups (low vs. high), respectively, confirmed significant association on all subscales, [depression: *X*^2^(1, N = 123) = 24.0, *p* < 0.001, *r* = 0.44; anxiety: *X*^2^(1, N = 123) = 10.8, *p* < 0.01, *r* = 0.30; stress: *X*^2^(1, N = 123) = 12.3, *p* < 0.001, *r* = 0.32].

**Table 3:** Descriptive statistics, severity levels, and COVID-19-related changes on the three subscales of the DASS-21, and for craving, displayed for the total sample and the two subgroups, respectively.

	**Total** **(***N*** = 123)**	**Low IES-R** **(***N*** = 90)**	**High IES-R** **(***N*** = 33)**
	**Mean (SD)/Frequency (percent)**	**Mean (SD)/Frequency (percent)**	**Mean (SD)/Frequency (percent)**
DASS-21 total score [0–63]	14.6 (13.7)	11.4 (13.2)	23.6 (10.8)
**Depression subscale**			
Depression score [0–21]	5.7 (5.6)	4.5 (5.3)	9.1 (4.9)
Normal (0–4)	65 (52.8%)	59 (56.6%)	6 (18.2%)
Mild (5, 6)	12 (9.8%)	9 (10.0%)	3 (9.1%)
Moderate (7–10)	23 (18.7%)	8 (8.9%)	15 (45.5%)
Severe (11–13)	8 (6.5%)	5 (5.6%)	3 (9.1%)
Extremely severe (14+)	15 (12.2%)	9 (10.0%)	6 (18.2%)
Change depression [−5 to +5]	+0.7 (1.4)	+0.5 (1.2)	+1.5 (1.7)
Improvement	5 (4.1%)	4 (4.4%)	1 (3.0%)
Deterioration	48 (39.0%)	27 (30.0%)	21 (63.3%)
No change	70 (56.9%)	59 (65.6%)	11 (33.3%)
**Anxiety subscale**			
Anxiety score [0–21]	3.5 (4.0)	2.6 (3.7)	5.8 (4.0)
Normal (0–3)	79 (64.2%)	67 (74.4%)	12 (36.4%)
Mild (4)	11 (8.9%)	6 (6.7%)	5 (15.2%)
Moderate (5–7)	14 (11.4%)	7 (7.8%)	7 (21.2%)
Severe (8, 9)	4 (3.3%)	3 (3.3%)	1 (3.0%)
Extremely severe (10+)	15 (12.2%)	7 (7.8%)	8 (24.2%)
Change anxiety [−5 to +5]	+0.9 (1.4)	+0.6 (1.2)	+1.7 (1.4)
Improvement	3 (2.4%)	2 (2.2%)	1 (3.0%)
Deterioration	52 (42.3%)	29 (32.2%)	23 (69.7%)
No change	68 (55.3%)	59 (65.6%)	9 (27.3%)
**Stress subscale**			
Stress score [0–21]	5.4 (5.0)	4.2 (4.9)	8.7 (4.1)
Normal (0–7)	86 (69.9%)	72 (80.0%)	14 (42.4%)
Mild (8, 9)	11 (8.9%)	6 (6.7%)	5 (15.2%)
Moderate (10–12)	15 (12.2%)	5 (5.6%)	10 (30.3%)
Severe (13–16)	6 (4.9%)	4 (4.4%)	2 (6.1%)
Extremely severe (17+)	5 (12.2%)	3 (3.3%)	2 (6.1%)
Change stress [−5 to +5]	+0.8 (1.4)	+0.5 (1.5)	+1.8 (2.6)
Improvement	14 (11.4%)	9 (10.0%)	5 (15.2%)
Deterioration	56 (45.5%)	34 (37.8%)	22 (66.7%)
No change	53 (43.1%)	47 (52.2%)	6 (18.2%)
**Craving**			
Craving [0–10]	2.9 (3.1)	2.7 (3.1)	3.6 (3.0)
Change craving [−5 to +5]	+0.4 (1.2)	+0.1 (0.9)	+1.1 (1.7)
Less craving	3 (2.4%)	3 (3.3%)	0 (0%)
More craving	21 (17.1%)	10 (11.1%)	11 (33.3%)
No change	99 (80.5%)	77 (85.6%)	22 (66.7%)

*SD, standard deviation; DASS-21, depression, anxiety, and stress scale−21 Item Version*.

#### Groupwise Comparisons for Low- and High-risk PTSD Groups

**Figure 1:**
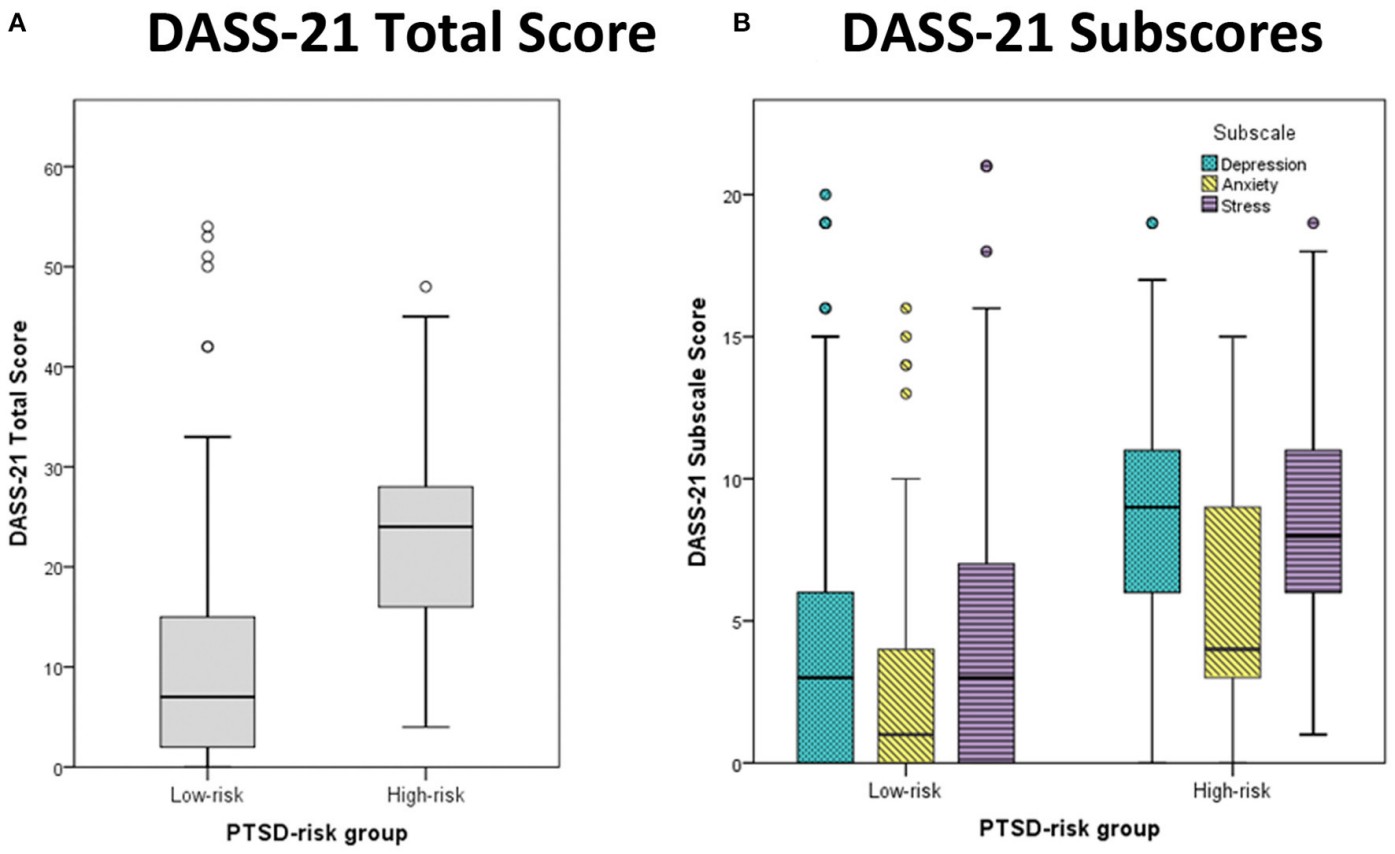
Boxplots for **(A)** the total score and **(B)** the three subscores of the DASS-21. Median scores are provided for the two PTSD-risk groups (left: low-risk, right: high-risk). The different subscale scores are depicted for depression (cyan), anxiety (yellow), and stress (purple). Outliers are presented as small circles.

Groupwise comparisons for the low- and high-risk PTSD groups for the total DASS-21 score, as well as for the three different subscores for depression, anxiety and stress were conducted using Mann Whitney tests (see Figure 1). Significantly higher scores were found for all the scales in the high-risk group, with all *p*s < 0.001, all *r*s > 0.41 (*z* = −5.2, *z* = −4.6, *z* = −4.8, *z* = −5.0, respectively).

#### Changes in Depression, Anxiety, and Stress

Changes in depression, anxiety and stress since the beginning of the pandemic were assessed on separate scales, with higher scores indicating a subjectively perceived worsening of the respective symptoms (e.g., ranging from −5 = “much less depressed” to +5 = “much more depressed”). Group differences between low- and high-risk PTSD groups were assessed using Mann Whitney tests for the three different subscales. Significantly higher scores were indicated by the high-risk PTSD group on all three subscales, for depression (*z* = −3.5, *p* < 0.001, *r* = 0.31), anxiety (*z* = −3.9, *p* < 0.001, *r* = 0.34), and stress (*z* = −3.3, *p* < 0.01, *r* = 0.29). These results assessing changes in depression, anxiety and stress indicate a more pronounced deterioration in symptoms for the high-risk PTSD group. Data on subjectively perceived deterioration, improvement or no change in depression, anxiety, and stress since the onset of the pandemic can be found in Table 3.

Taking the results together, the high-risk PTSD group indicated not only stronger subjective clinical symptoms of depression, anxiety and stress, but also a more pronounced decline in the symptomatology. In fact, roughly a third of our patients in the high-risk PTSD group reported a deterioration on all three scales.

#### Craving

In respect to craving (scale from 0 to 10), a Mann Whitney test revealed no significant difference between low- and high-risk PTSD groups (*p* = 0.108, *r* = 0.14), but a significant increase in craving since the beginning of the pandemic for the high-risk group (*z* = −3.2, *p* < 0.01, *r* = 0.20; see Table 3 for more details). Noteworthy, one third of the patients in the high-risk group reported an increase in craving in this time period.

Please use a small indention for ALL of the following rows for all the subscales (see also original submission):

### Concomitant Use and the Austrian Drug Market

In our total sample of patients in OST, 48% reported concomitant use of non-prescribed illicit substances. A qui-square test of independence was conducted to evaluate potential associations between risk for PTSD (low- vs. high-risk) and concomitant use (no vs. yes). Results confirmed a significant association between the two variables, *X*^2^(1, N = 123) = 4.4, *p* < 0.05, *r* = 0.19. This finding suggests that those reporting concomitant use were also more likely to be part of the high-risk PTSD group.

Among this group indicating concomitant use (*N* = 59, see Table 4 for descriptive statistics), most reported consuming cannabis (80%), followed by heroin (24%), cocaine (17%), and unprescribed benzodiazepines (15%). Only a small proportion indicated consumption of methamphetamines (3%), hallucinogens (3%), or amphetamines (2%). In respect to legal substances, 17% indicated drinking alcohol regularly and 86% in this group were smokers. The subgroup of patients reporting concomitant use illicit drugs had a mean score of 9.23 (*SD* = 3.1) on the DUDIT-C, and indicated no relevant changes in frequency (mean = −0.1) and quantity of consumption (mean = −0.3) Developments on the Austrian drug market were evaluated for pricing, availability problems and quality of illegally purchased substances (on scales from −5 to +5). Although about 15% of these patients reported an increase in both, prices and availability problems, the majority indicated no change on the three scales, suggesting a rather stable situation on the Austrian drug market. In sum, no substantial changes could be detected based on the patients’ responses, with only slight increases in prices (mean = 0.2) and availability problems (mean = 0.8), and a mean decrease in quality (mean = −0.7).

**Table 4:** Descriptive statistics for changes in consumption patterns (frequency, quantity) and variables regarding changes on the Austrian drug market (prices, availability, and quality) are displayed for the subsample of patients indicating concomitant use of illicit substances (*N* = 59).

		**Frequency (percent)**		
***Scales*** **[−5 to +5]**	**Mean (SD)**	**Less/lower**	**No change**	**More/higher**
Change frequency	−0.1 (1.7)	10 (16.9%)	43 (72.9%)	6 (10.2%)
Change quantity	−0.3 (1.5)	12 (20.3%)	42 (71.2%)	5 (8.5%)
Change prices	+0.2 (1.5)	3 (5.1%)	47 (79.7%)	9 (15.3%) ^*^
Change availability problems	+0.9 (1.6)	1 (1.7%)	36 (61.0%)	19 (16.9%) ^*^
Change quality	−0.7 (1.6)	15 (25.4%)	39 (66.1%)	2 (3.4%) ^*^

* *Percentages do not add up to 100% due to missing values (N.A.) on these scales. SD, standard deviation*.

One third (*N* = 41) of patients in our total sample had an additional prescription of benzodiazepines. A qui-square test of independence did not result in a significant association between risk for PTSD (low- vs. high-risk) and benzodiazepine prescription (no vs. yes), *X*^2^(1, N = 123) = 0.2, *p* = 0.67, *r* = 0.04. This finding suggests, that patients with an additional prescription of benzodiazepine were not at higher risk for PTSD due to the pandemic.

## Discussion

Concerns about the negative impact of the COVID-19 pandemic on mental health of the SUD population have been raised by experts early on. Since then, many studies have focused on investigating these consequences in terms of clinical symptoms like PTSD, depression and anxiety. However, given the sudden and unexpected onset of the pandemic, classical comparisons of results before and after the beginning of the crisis fell short. Hence, findings are often difficult to be directly associated to the impact of the pandemic itself. In the current study, we aimed to overcome this shortcoming by assessing risk for PTSD directly linked to the COVID-19 pandemic and no other traumatic event. Furthermore, self-reported changes in symptomatology of depression, anxiety and stress, as well as changes in consumption patterns and at the Austrian drug market were evaluated.

The IES-R was adapted to measure PTSD symptoms due to the COVID-19 pandemic itself [similar to Vanaken et al. (16)]. By applying the recommended cutoff-score for patients with SUDs (42), we found that more than a quarter (27%) of our patient in OST developed an elevated risk for PTSD. However, this risk for PTSD as assessed by the IES-R score has to be interpreted with caution, since it does not fulfill the same clinical criteria for a diagnosis made by a specialist. Our results of a binary logistic regression analysis further indicate that self-reported higher negative impact by psychological and economic COVID-19-related aspects increase the risk to develop PTSD. Psychological burden in this study summarized perceived stressed and isolation and the pandemics’ impact on mental health as feelings of irritability, depression, anger or helplessness. Similar findings were reported by an Italian study, which confirmed a significantly negative association between well-being with depressive, anxious and PTSD symptoms (23), as well as an Chinese study reporting a moderate-to-severe psychological impact during the initial phase of the pandemic (24). The individually perceived burden in terms of negative financial consequences, often resulting in income cuts due to short work or even job loss, were rated on the scale for economic COVID-19 factors. The finding of economic factors as a second significant risk factor in our model is in line with a study identifying lower perceived economic stability as one risk factor for PTSD during the pandemic (15).

In general, rather high scores of psychopathological symptoms of depression, anxiety, and stress were observed in our sample of patients in OST. For depression, half of our participants indicated scores above exceeding the normal severity level, and nearly 40% indicated a deterioration in these symptoms since the beginning of the pandemic. Prevalence of current depression in a sample of patients with OUD were reported up to 32% (with reports up to for 75% lifetime prevalence) before the pandemic (20). Our findings exceed this estimated incidence, but can be explained by the high percentage of patients indicating a worsening of depressive symptoms, which is also in line with other studies on the current pandemic. Similar findings were observed for anxiety and stress, with approximately one third scoring above the normal level, and more than 40% reporting a deterioration of these symptoms. All of these negative consequences were anticipated by experts early on, and have already been confirmed by several studies (13, 23). In our study, pairwise comparisons of groups with low and high risk for PTSD, respectively, confirmed the differences in depression, anxiety and stress levels. Importantly, the high-risk PTSD group also reported a more pronounced increase of these symptoms since the onset of the pandemic. These findings affirm the expected negative impact on mental health of patients in OST and further contribute to identify a risk group of patients, who should receive special attention in health care during this ongoing pandemic.

Comorbid psychiatric disorders can crucially impact treatment outcome of patients suffering from opioid use disorder (OUD) (20). In this context, the complex interactions between depression and substance use disorders are highlighted. While the important role of concomitant treatment of depression in alcohol use disorder is well-documented, the impact of depression on OUD treatment remains unclear (20). Our findings underline the importance of depressive symptoms among patients in opioid substitution therapy (OST). Especially the significantly higher scores on the depression, anxiety, and stress subscales, alongside the more severe self-reported deterioration in the high-risk PTSD group call for a closer look at these comorbid mental disorders during this ongoing pandemic. Short screening instruments for these symptoms are available, and adapted interpretation of scoring for populations suffering from SUDs have already been put forward for some of them, like the IES-R (42). The DASS-21 was identified as a suitable screening tool for depression in an SUD population when administered after detoxification (49). The current pandemic calls for a further adaptation of existing tool (16) to specifically determine the current effect. These modifications might allow to rapidly and effectively screen for symptoms, which have been identified to provide a particular source of distress for this population during COVID-19. Integrating these screenings into medical history taking might be a successful way to identify especially vulnerable individuals and potentially counteract the pandemic as a potential additional reason for early dropout in OST.

Concomitant use of illicit drugs among patients in OST is a well-acknowledged and still an important topic in addiction research. Among our sample nearly half of the patients admitted consumption of other substances than prescribed. This is an extension to our previous research based on a small sample of patients suffering from SUDs (7). Crucially, in our prior sample nearly 80% had admitted concomitant use of illicit drugs, which might be explained by the fact, that this prior sample consisted mainly of inpatient participants, and not all of them were in OST. Notably, in the current study a significant association between concomitant use and high risk for PTSD was found. This finding should raise concern about patients indicating concomitant use during the pandemic, since they might also be at higher risk for PTSD.

Substantial changes on the Austrian drug market—in particular for quality, prices, and availability of illicit substances—were not observed in this study. This finding is an extension of our prior research, in which a rather stable situation was indicated during the initial phase of the pandemic (7). Prior research on the impact of the pandemic on addictive behaviors indicated both, decrease and increase in substance use, with different samples and consumption patterns. In this context, different prevention strategies depending on the severity of substance use were recommended (12). In the current study, our findings did not reflect any noteworthy changes in drug consumption in terms of frequency and quantity of substance use.

This study has some limitation. First, the sample in this cross-sectional study was approached at our outpatient facilities, and a selection bias cannot be completely excluded. In this context, the reported changes in symptoms due to the pandemic were also assessed cross-sectionally. Second, our results are solely based on patients’ self-reports, whereas no professional evaluation of the psychiatric symptoms were assessed for this study. Importantly, the risk for PTSD in this study was based on the IES-R total score and not on a professional evaluation. Furthermore, the symptoms assessed by the IES-R and DASS-21 might overlap to a certain degree. A clear distinction between the different symptoms as well as a diagnosis of a clinically relevant disorder is not within the scope of this study. Additionally, we want to emphasize that individual drug use and consumption patterns in this study were subjectively reported by the patients and not measured objectively. Future studies should additionally explore the impact of the pandemic on the development of PTSD as a professional diagnosis, and include objective measures of drug use. Third, this study is based on current short-term findings, and long-term observations and developments have to be closely monitored.

Nonetheless, this study investigated a sample of patients in OST, and contributed to existing literature by findings on the impact of the pandemic on a particularly vulnerable group of patients. Further adaptations of well-established screening tools for psychiatric comorbidities to this subpopulation and the current pandemic is recommended. Based on our results, identification of particularly vulnerable individuals might be helpful for health care professionals to counteract to the potential rise of PTSD and depression in this population during this ongoing pandemic.

## Data Availability Statement

The raw data supporting the conclusions of this article will be made available by the authors, without undue reservation.

## Ethics Statement

The studies involving human participants were reviewed and approved by Ethics Commission of the Medical Faculty of the Johannes Kepler University Linz. The patients/participants provided their written informed consent to participate in this study.

## Author Contributions

IF-L: conceptualization, formal analysis, methodology, and writing—original draft preparation. KY: conceptualization, resources, and writing—review. NG: conceptualization and writing—review. MT, S-TG, and AS: data curation and writing—review. JR: conceptualization, data preparation, writing—original draft preparation, and review. All authors have read and approved the final manuscript.

## Conflict of Interest

The authors declare that the research was conducted in the absence of any commercial or financial relationships that could be construed as a potential conflict of interest.

## Publisher's Note

All claims expressed in this article are solely those of the authors and do not necessarily represent those of their affiliated organizations, or those of the publisher, the editors and the reviewers. Any product that may be evaluated in this article, or claim that may be made by its manufacturer, is not guaranteed or endorsed by the publisher.

## Footnotes

^1^DASS-21 scores and subscores are sometimes multiplied by a factor 2, in order to match the scoring of the original 42 items version. This should be taken into account, when comparing with other studies. Levels of severity on the subscales for depression, anxiety, and stress were accordingly adjusted to the recommended cutoff scores in the descriptive analysis and Table 2.
